# Overcoming intra-molecular repulsions in PEDTT by sulphate counter-ion

**DOI:** 10.1080/14686996.2021.1961311

**Published:** 2021-12-23

**Authors:** Dominik Farka, Theresia Greunz, Cigdem Yumusak, Christoph Cobet, Cezarina Cela Mardare, David Stifter, Achim Walter Hassel, Markus C. Scharber, Niyazi Serdar Sariciftci

**Affiliations:** a Linz Institute for Organic Solar Cells (LIOS) Physical Chemistry, Johannes Kepler University Linz, Linz, Austria; b Institute of Solid State Physics, Johannes Kepler University-Linz, Linz, Austria; c Institute of Chemical Technology of Inorganic Materials (TIM), Johannes Kepler University Linz, Linz, Austria; d Center for Surface and Nanoanalytics (ZONA), Johannes Kepler University Linz, Linz, Austria; e Linz School of Education, Johannes Kepler University Linz, Linz, Austria; f Center of Chemistry and Physics of Materials, Faculty of Medicine/Dental Medicine, Danube Private University, Krems, Austria; g Christian Doppler Laboratory for Combinatorial Oxide Chemistry (COMBOX), The Institute of Chemical Technology of Inorganic Materials (TIM), Johannes Kepler University Linz, Linz, Austria

**Keywords:** PEDOT, PEDTT, conducting polymers, magnetotransport, metal–insulator transition, 105 Low-Dimension (1D/2D) materials, 106 Metallic materials, 201 Electronics / Semiconductor / TCOs, 203 Magnetics / Spintronics / Superconductors, 301 Chemical syntheses / processing, 500 Characterization

## Abstract

We set out to demonstrate the development of a highly conductive polymer based on poly-(3,4-ethylenedithia thiophene) (PEDTT), PEDOTs structural analogue historically notorious for structural disorder and limited conductivities. The caveat therein was previously described to lie in intra-molecular repulsions. We demonstrate how a tremendous >2600-fold improvement in conductivity and metallic features, such as magnetoconductivity can be achieved. This is achieved through a careful choice of the counter-ion (sulphate) and the use of oxidative chemical vapour deposition (oCVD). It is shown that high structural order on the molecular level was established and the formation of crystallites tens of nanometres in size was achieved. We infer that the sulphate ions therein intercalate between the polymer chains, thus forming densely packed crystals of planar molecules with extended π-systems. Consequently, room-temperature conductivities of above 1000 S cm^−1^ are achieved, challenging those of conventional PEDOT:PSS. The material is in the critical regime of the metal–insulator transition.

## GRAPHICAL ABSTRACTS

**Figure:**
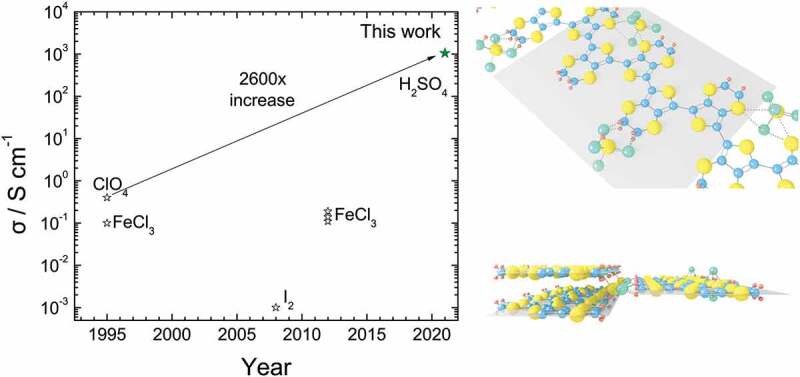

## Introduction

The rising importance of conductive polymers is beyond discussion: (lightweight) solar applications [1–4], biointegration [5], pressure sensors [6], and actuators [7], as well as thermoelectric applications [8] and batteries [9] were demonstrated. Beyond diversity in application, high conductivities (>6000 S cm^−1^) [10–13] challenging those of inorganic materials [14], and even semi-metallic [15,16] and metallic properties [17–21] were shown to be possible. To this date, however, the portfolio of high-end conductive polymers remains limited. Design rules for good conductors are concerned with molecular (polymer) architecture [22,23] and development of disorder-suppressing processing techniques [24–27]. An excellent review and comprehensive introduction to conductive polymers was published recently [28]. In this context, the role of the counter-ion typically receives little attention [12,17,29,30] beyond stabilizing the charge-balance while introducing mobile charge carriers.

**Figure 1.**
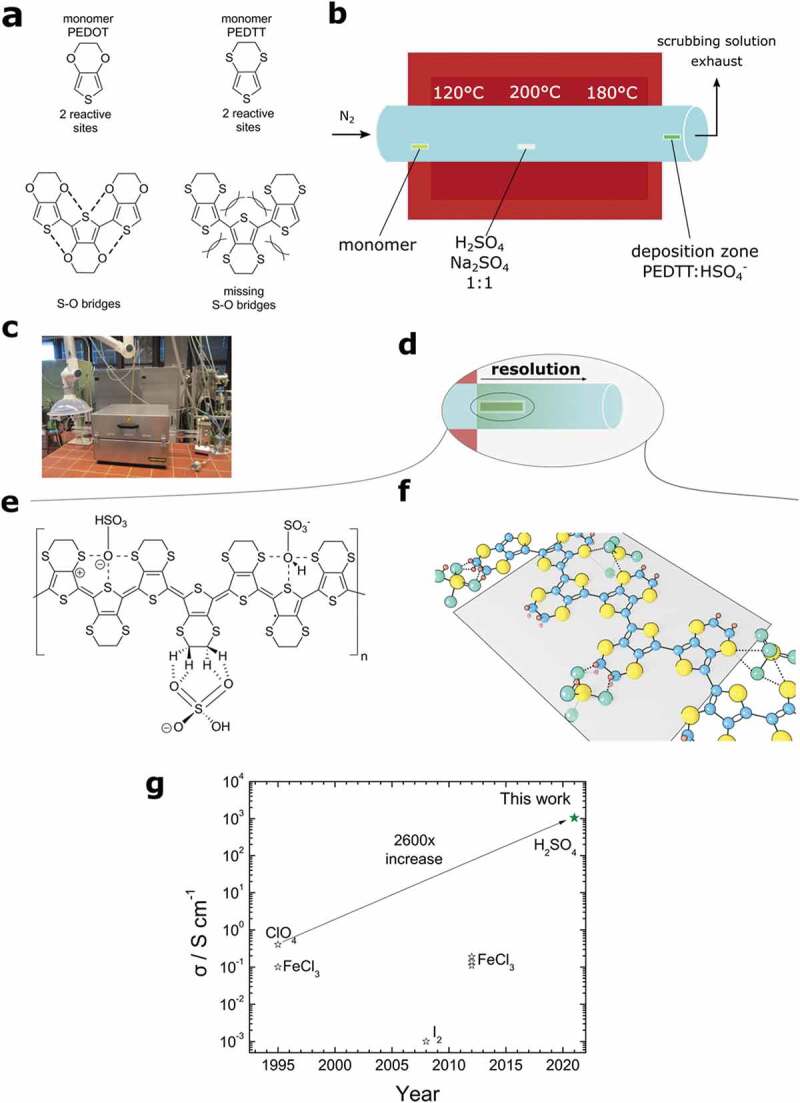
Rationale of how to boost conductivity in PEDTT. (a) Intramolecular interactions in PEDOT and PEDTT. The thioethers in PEDTT show repulsion with respect to the thiophene’s sulphur atom, resulting in inferior transport properties. (b) Sketch of the tube furnace used to synthesize PEDTT:sulf (PEDTT:HSO_4_^−^) samples with positions of the sample and reactants. (c) Tube furnace used in this work. (d) Illustration of the material resolution found in our oCVD-method. At the end of the tube furnace, the deposition zone commences: As a temperature gradient forms, the material with the highest conductivity was found to be deposited first. A substrate situated just at the end will be coated with highly conductive PEDTT. (e) The deposition method in tandem with the small counter-ion, we hypothesize, will intercalate the conductive polymer. The possible interactions of polymer and counter-ion are illustrated and based on previous work [33–35]. (f) A hypothetic 3D-model of the PEDTT based on the proposed interactions, assuming a flat conformation with an extended π-system (grey plane) just as suggested by Massonnet et al. for PEDOT:trifluromethane sulfonate [17]. (g) The history of conductivities achieved in PEDTT. The conductivity achieved in PEDTT: sulphate beat the previous record by 2600-fold. Note, that then also an oxide in the form of ClO_4_^−^ was used as a counter-ion, supporting our hypothesis that a molecular-interaction drives the adoption of a planar conformation [31, 37]

Herein, we demonstrate the development of a highly conductive poly-(3,4-ethylenedithia thiophene) (PEDTT; Figure 1) based material with temperature-independent transport at low temperatures.

PEDTT [31] is the structural all-sulphur analogue of poly-(3,4-ethylenedioxy thiophene) (PEDOT), a conductive polymer that has generated substantial research interest by its outstanding optoelectronic properties [2,10,11,18,28,30,32]. One of the reasons for PEDOT’s electronic performance lays the present intra-molecular interactions, which was illustrated by studies of PEDTT and its derivatives [23,33–35]. Both polymers consist of a conjugated thiophene ring fused to a six-membered ring containing either oxygen or sulphur in the 6 and 9 positions. The change from oxygen to sulphur is sufficient to either yield a system with self-rigidifying structural properties (PEDOT) or a system that is prone to disorder due to intra-molecular repulsions (PEDTT). These repulsions arise from sulphur–sulphur (S–S) interactions between the thiophene-ring and the thioethers of the side ring. In PEDOT, on the contrary, attractive sulphur-oxygen (S–O) interactions lead to a self-rigidification and high conductivities are fairly easily established [23,33–35]. The conclusion of these studies was that PEDTT is unable to show high conductivities because this tendency to disorder would always result in localization of charge carriers [33–35]. And so far, their statements remained true as to date, PEDTT is notorious for conductivities below 1 S cm^−1^ [31,36,37], despite multiple attempts to improve its conductivity.

In order to achieve high conductivities in PEDTT, we hypothesize that it is necessary to establish rigidifying structural properties by positive, intra-molecular interactions. Albeit attractive S–S interactions are known in literature, these are known in aliphatic, non-cyclic systems, where for steric reasons S–S non-covalent attractions are possible [38–40]. Therefore, we needed an external influence, such as the addition of an adequate counter-ion.

Our choice fell on sulphate as the counter-ion, as it is supplies oxygen to interact with the polymer backbone, thus possibly mimicking the structural properties found in PEDOT. Further, it was shown to be a potent doping agent for conductive polymers [41,42], and a synthesis method for highly conductive PEDOT with said counter-ion was already developed previously [23,43].

Starting from the monomer and sulphuric acid, the polymer was grown on top of sapphire substrates and simultaneously doped by sulphate-ions using oxidative chemical vapour deposition (oCVD) [4,9,44–47], as depicted in Figure 1b,c. The advantageous use of sulphate ions in the context of doping conductive polymers has been previously demonstrated by Genz and Lohrengel [41,42] and was also shown by Massonnet et al. for PEDOT [17,44].

As a technique, the strengths of oCVD are well known and was demonstrated to have significant advantages in the context of PEDOT [11,45]. In our case, the linear flow of carrier gas adds an additional feature: intrinsic purification and material resolution akin to gas chromatography (Figure 1d). Consequently, even the resulting polymer is not deposited in equal quality across the deposition zone, and the substrate-position becomes a crucial parameter to optimize. In our case, the substrate had to be placed just at the end of the tube furnace, as illustrated in Figure 1b. In the case of PEDOT:sulphate, this synthesis method was demonstrated to deliver semi-crystalline material with outstanding electrical properties [21,32].

The interactions between the counter-ion and PEDTT, which moderated the intra-molecular repulsions of the latter and culminated in great conductive performance, are illustrated in Figure 1e,f.

We support our counter-ion hypothesis by comparing drop-cast films of PEDTT doped with sulphuric acid (H_2_SO_4_) and gold(III)chloride (auric chloride, Au_2_Cl_6_). The reduction of sulphuric acid has a lower electrochemical potential than reduction of the chloride [49]; consequently, it is a weaker doping agent. Despite this, sulphate-doped films showed higher conductivities, and remained conductive down to 160 K. This suggests that resulting film suffers less from disorder when doped with a sulphate counter-ion, which was further confirmed by X-ray diffraction (XRD) measurement, showing crystalline peaks in the acid-doped film, only, hinting at an intrinsic advantage of the used dopant in agreement with literature [41,42].

XRD-measurements on oCVD-grown PEDTT (further denoted as PEDTT:sulf) reveal the formation of crystallites tens of nanometres in size, which result in a tremendous improvement in conductivity of >2600-fold conductivity increase at room temperature, relative to the previous record [31] (Figure 1g). Even at low temperatures (3.6 K limited by the cooling stage) a finite conductivity of an impressive 50 S cm^−1^ was retained. The material exhibits positive magnetoconductivity (MC) and is found to be in the critical regime of the metal–insulator transition.

## Experimental

Synthesis and deposition of the polymer were performed according to a previously reported oCVD procedure [32]. The monomer was obtained from the company ‘1-Material’ and is a yellowish powder. As it is not a liquid, it is necessary to slightly heat the material in order to melt it and allow it to participate in the gas phase reaction (Table 1). It was found to be ideal to place the vial containing the monomer just at the beginning of the first heated zone (120°C). The exact position has to be found iteratively for each furnace, depending on tube-diameter, tube-thickness, and relative carrier-gas-flow-velocity. The material with the highest conductivity was found just in the beginning of the deposition zone (relative to the gas-flow). The reaction chamber takes several minutes to reach optimal reaction conditions; a non-linear time dependence growth-rate results. A 60 nm thin film would be deposited in 45 minutes (1.33 nm min^−1^), 400 nm thick samples in 105 minutes (3.8 nm min^−1^). The synthesis was optimized for maximal conductivity until reproducibility in tens of samples was ensured in two-probe conductance measurements, before conductivity measurements on a cooled four-probe stage were performed.

**Table 1.** Synthesis parameters for PEDTT in three-zone furnace. ‘Insulation (Exit)’ indicates an 8 cm long zone where the thermal-insulation of the tube furnace touches the glass-tube, where a relatively mild thermal gradient is found

	Heating zone 1	Heating zone 2	Heating zone 3	Insulation (exit)
Temperature/°C	120	200	180	170–180
Added components	Monomer	Oxidant	None	Substrate (deposition)

For a single heating-zone furnace, 200°C should be used; the positioning of the monomer-containing vial and the substrate must be chosen carefully at the tube entrance and exit, respectively. The use of additional insulation, *i.e*. aluminium foil to extend the deposition zone and thus improve material-quality resolution, is highly recommended. All syntheses were conducted at ambient pressure.

Drop-cast films of PEDTT (1-Material) were prepared from 3.5 mg mL^−1^ solutions in THF – 10 µL were dropped onto sapphire substrates. The formed films and the doping agent were heated using a heat plate (80°C).

For sulphuric acid doped films, the substrates were coated by 20 µL of the concentrated acid and left for 10 min on the heating plate. In order to remove superfluous acid, a spin-coater was used (as described elsewhere [20]). In our case, 2-propanol followed by two steps of deionised water was employed. This procedure is gentler and prevents delamination.

In order to dope PEDTT with gold chloride, the dopant was dissolved in acetonitrile and heated to 80°C. Doping was done by immersion of the films into the solution followed by rinsing with acetonitrile. All steps were performed in a glove-box under nitrogen atmosphere.

X-ray photoelectron spectroscopy (XPS) was performed on a Theta Probe system from Thermo Fisher Scientific (East Grinstead/UK), which is equipped with a monochromatic AlK_α_ X-ray source (1486.7 eV). Glass substrates with Cr/Au (8 nm and 80 nm, respectively) evaporated prior to PEDTT deposition were used. XPS survey scans as well as high-resolution C1s, O1s, S2p spectra were acquired from films with nominal thickness of 30 nm. The S2p levels were employed to calculate the stoichiometry of PEDTT and sulphate ions, respectively. The S2p data reveals two clearly distinguishable contributions arising from the PEDTT and the corresponding counter-ion.

Initial optical properties were measured using UV-vis absorption and reflection spectroscopy (Perkin Elmer Lambda 1050) as well as ATR-FTIR (Bruker Vertex 80). For the latter, the transmission data were recalculated in the following way

$$-\frac{\Delta T}{T}=-\frac{T_{polymer}-T_{glass}}{T_{glass}}\approx Abs.$$

to give a value corresponding to the absorption+reflection.

The films were also characterized by variable-angle, spectroscopic ellipsometry (VASE, Woollam M-2000) with rotating compensator ellipsometer, and modelled using the corresponding VASE programme. Fitting was done using a B-spline representation of the imaginary part of the dielectric function. The real part of the dielectric function is calculated in this procedure by means of the Kramers–Kronig relations which ensures a physically consistent line shape of both components.

Electrical transport and magneto transport properties were determined using DynaCool PPMS and Lakeshore 8400 series. Sapphire substrates with evaporated Cr/Au (8/80 nm) were coated with PEDTT:sulf. For the DynaCool PPMS, 4-probe contact geometry was used, observing a temperature range between 2 and 300 K and a magnetic field between ±9 T. For the Lakeshore system, van-der-Pauw (vdP) geometries were employed, investigating a temperature range of 10–300 K. As the vdP-geometry allows for electrically switching between the contacts, it enabled us to eliminate the geometric factors except the sample thickness, minimizing the experimental error in conductivity measurements.

All X-ray diffraction (XRD) measurements were performed on a Philips-Pro X’Pert system working in Bragg–Brentano geometry with CuK_α_ radiation.

Following Braggs law [49]

2dsinθ=nλ

the stacking distance was calculated, where *d* is the stacking distance, *θ* the incidence angle relative to the plane of stacking, *n* is the diffraction order, and *λ* the wavelength of the CuK_α_ radiation.

Further, through Scherrer’s formula

$$L=\frac{0.9\cdot \lambda }{FWHM\cdot cos\left(\frac{2\theta }{2}\right)}$$

where *L* is the crystallite size and FWHM the peaks full width at half maximum, the crystal-size can be estimated.

All 3D illustrations were done using Blender 2.79b software, taking into account literature values for bond length and stacking distances in agreement with our measurements. Colour code of spheres: H ≡ red; C ≡ blue; O ≡ turquoise; S ≡ yellow.

## Results and discussion

**Figure 2.**
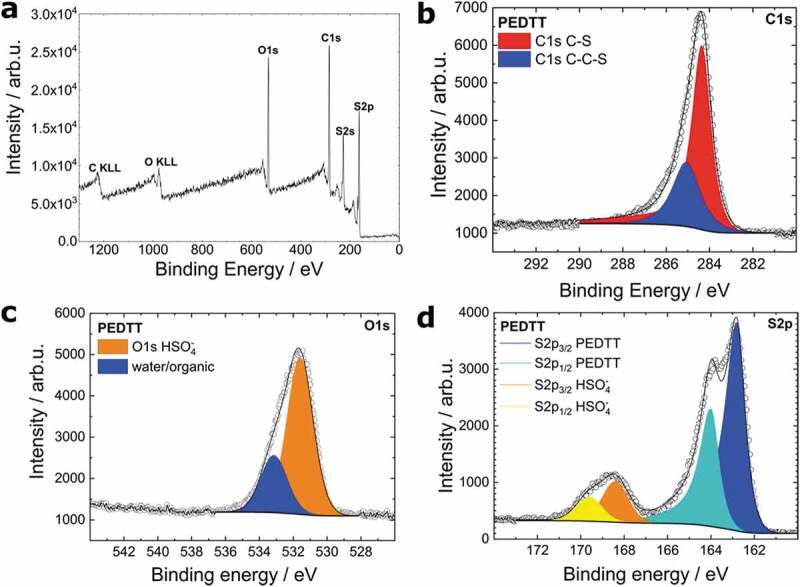
(a)The XPS survey spectrum confirms the presence of carbon, oxygen, and sulphur. (b) The XPS high resolution C1s spectrum reveals the presence of two carbon species referring to the chemical situation in PEDTT. (c) The XPS high resolution O1s spectrum of oCVD-grown PEDTT:sulf. of oCVD-grown PEDTT:sulf. The orange peak originates from the sulphate, the blue form a not further defined component, possibly water. (d) S2p XPS spectrum of the same sample. The ratio of PEDTT-to-sulphate indicates an extraordinary doping ratio of 1.6:1. We conclude that this is an exaggerated value resulting from sulphuric acid strongly adsorbed to the surface

**Figure 3.**
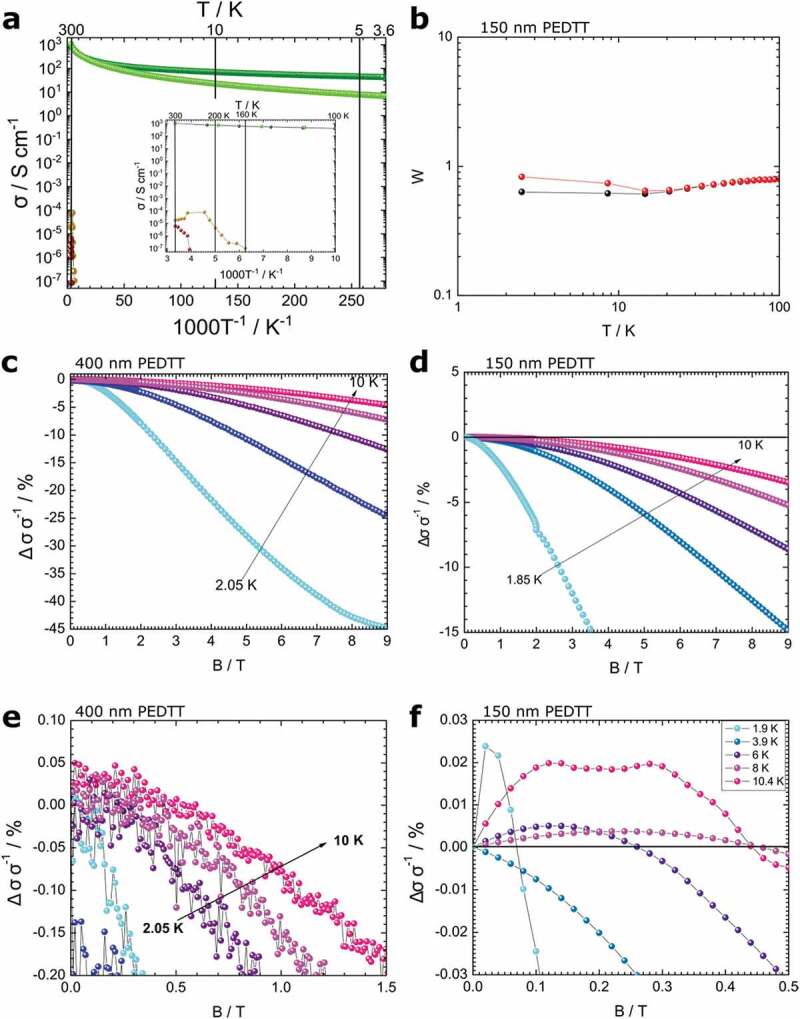
(a) Conductivity plots for several PEDTT samples. Sulphate-containing materials deliver the highest conductivity, the thinnest oCVD-grown film delivers the best performance overall. Inset: Enlarged conductivity plot with focus on the drop-cast materials. The auric chloride doped material is outperformed by the sulphate-doped counterpart despite sulphuric acid being the weaker oxidant. An effect of the counter-ion is observed. (colour code: dark green≡ 150 nm oCVD PEDTT; bright green ≡ 400 nm oCVD PEDTT; ochre ≡ sulfuric acid drop-cast PEDTT; crimson ≡ auric chloride drop-cast PEDTT. (b) W-plot of the films characterized in (d,f). The material is in the critical regime of the MIT in the absence of a magnetic field. At 9 T, these properties are suppressed and an insulating system is established. (c) MC measurement of 400 nm thick oCVD-grown PEDTT at temperatures between 1.85 K and 10 K. (d) MC measurement of 150 nm thick oCVD-grown PEDTT at temperatures between 2.05 K and 10 K. (e) Enlarged results of (c). Magnetolocalization is clearly visible. If magneto-conductance is present, it cannot be distinguished over the noise. (f) Enlarged results of (d). An interplay of magnetolocalization and MC can be seen, indicating a metallic system according to Menon et al. [68]

To identify the material as the polymer, XPS (Figure 2) and FTIR (Figure 3a) were measured.

Through XPS, we gained insight about elemental composition, monomer integrity, and the doping level.

The survey scan in Figure 2a confirms the elemental purity of the material, a property essential for extraordinary conductivity [32]. Further, high-resolution spectra were therefore measured for carbon, sulphur, and oxygen (Figure 2b-d).

The C1s spectrum (Figure 2b) is fitted with two peaks, referring to two distinct carbon species that are bound to sulphur. The peak at 284.4 eV (70.1 at.%) is ascribed to the carbon in the conjugated part [50], while the less intense peak (29.9 at.%) at the higher binding energy side is annotated to the non-conjugated protection group, which is similar to the C-C-S contribution presented in the XPS-database by G. Beamson, D. Briggs, and Surface Spectra Ltd [51]. Their relative intensities are in agreement with the expected values, indicating that the monomer polymerizes with little to no side-reactions, *i.e*. the polymerization occurs exclusively in the positions 2 and 5 of the thiophene ring. First and foremost, this indicates that the material does not decompose in the reactive environment of the tube furnace.

The O1s spectrum (Figure 2c) suggests that oxygen is present in two forms: the first one corresponds to sulphuric acid, whereas the second originates from either water or a further not specified organic component. Sulphur is expected to be present in the polymer and sulphuric acid, which is in good agreement with the XPS measurement (Figure 2d). By combining the O1s and the S2p spectra, we determined the doping ratio to be 1.6:1 in the favour of polymer-subunits: This ratio is well above any expected doping ratio and must be considered adsorbed sulphuric acid rather than an actual doping ratio. Due to the hygroscopic nature of sulphuric acid, we suggest that the unknown oxygen-signal in the O1s spectrum comes from adsorbed water. Based on doping stoichiometry, conductivity, and the findings reported by Massonnet et al. on sulphuric acid doped PEDOT, we believe that the doping species is HSO_4_^−^ [52].

To deliver a second proof for the chemical identity of the film and to investigate the materials potential for use in light-emitting diodes (LED) or solar cells optical measurements were conducted (Figure 3a, b).

In the infrared region around 3500 cm^−1^ (Figure 3a), a weak contribution of water and sulphuric acid can be seen. This indicates that both species, especially the high amounts of sulphuric acid observed in the XPS, are possibly adsorbed to the surface and therefore are not distributed throughout the sample. This also means that the doping ratio derived by XPS is vastly exaggerated by the surface sensitivity of the method. The presence of protonated sulphate can be further seen in the strong absorption between 1200 and 1300 cm^−1^. The fingerprint region is in good agreement with previous publications concerning the material, confirming that the synthesis method yields the material claimed [53,54].

The UV-vis measurements (Figure 3 b) of the material indicate a transparent material with the lowest absorption in the green spectral region. This suggests a possible application in light-emitting diodes.

The films were also characterized by variable-angle of incidence (55°, 60°, 65°, 70°, and 75°), spectroscopic ellipsometry (VASE, Woollam M-2000) with rotating compensator ellipsometer) and modelled using the corresponding VASE programme (Figure 3 c). Measurements in different spots of the material yielded results in good agreement with each other. Fitting was done using a B-spline representation of the imaginary part of the dielectric function of the film on the glass substrate. The dielectric function of the latter substrate material was determined separately. The real part of the dielectric function is calculated in this procedure by means of the Kramers–Kronig relations, which ensures a physically consistent line shape of both components. The electronic origin of the line shape is not analyzed in this way. However, we would note that the increase in the real as well as the imaginary part of the dielectric function towards the IR is not simply explainable by free charge carriers *i.e*. a Drude response function.

Below 0.18 eV, a strong excitation of the glass-phonons utterly superimposes the contribution of the material. A clear separation is therefore not possible. Simulations of roughness revealed a mean roughness of 8 nm for the nominally 26 nm thick material. This might appear drastic, yet it correlates well with XRD results and previous findings in PEDOT:sulphate. At such low thicknesses, an archipelago-like structure forms where crystallites larger than the mean thickness of the film are surrounded by a thinner, disordered material.

These structural thin-film properties could also be observed in charge-carrier transport measurements in the temperature range between 2 or 3.6 and 300 K were performed (Figure 3). The lower temperature boundary was given by the technical boundaries of the cryo-platform at the time of measurement.

**Figure 4.**
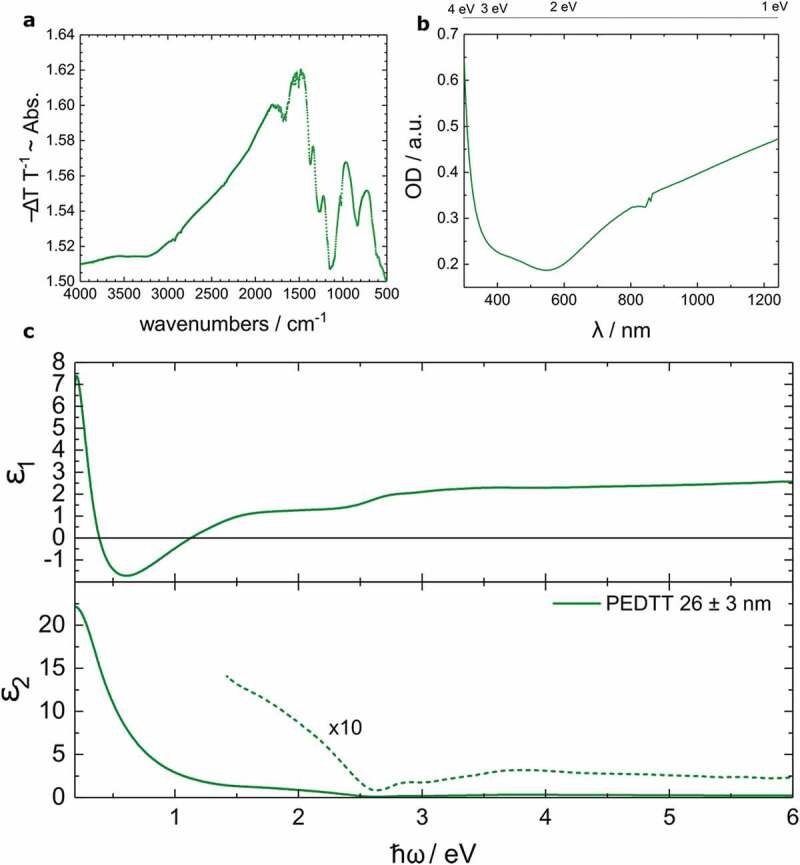
(a) ATR-FTIR measurements of oCVD PEDTT:sulphate (80 nm) on glass. The spectrum is in good agreement with the findings of Cravino et al. [56]. (b) UV-vis measurements of the substrate presented in (a). The material is transparent and exhibits a minimum of absorption in the green spectral region. (c) VASE spectrum of 26 nm PEDTT:sulf. The material is doped and possibly forms out an archipelago-like structure, similar to the one found for PEDOT:sulphate [21]

Films of 400 nm and 150 nm of oCVD-grown PEDTT:sulf were compared with drop-cast films of PEDTT doped with sulphuric acid (beige dots) and gold(III)chloride (crimson dots), respectively (Figure 4a).

The room temperature conductivity of PEDTT:sulf was determined to be 1050 S cm^−1^. This presents a significant achievement in itself, as it outperforms the previous record from 1995 by more than 3 orders of magnitude (>2600-fold) [31] at room temperature. Both samples exhibit finite conductivities at low temperatures (3.6 K, see Table 2). This suggests that the conductivity is determined by a temperature independent conduction mechanism at low and a hopping, thermally activated transport mechanism at high temperatures. We calculated the activation energy, E_a_ [55], for the hopping transport in the 150 nm and the 400 nm PEDTT:sulf thin-films, resulting in 10.88 and 9.22 meV, respectively (Table 3). We believe that the difference in the two values comes from the prevalence of temperature-independent transport mechanisms dominating in the thinner sample, thereby seemingly resulting in the higher activation energy. In both cases, the value lies well below the thermal activation energy (~26 meV) at room temperature [55].

**Table 2.** Conductivity retention of oCVD grown films (400 and 150 nm thick) at 3.6 and 300 K

Thickness/nm	σ _300 K/_S cm^−1^	σ_3.6 K_/ S cm^−1^	100*σ_3.6 K_ (σ_300 K_)^−1^/ %
400	1050	10	0.95
150	1050	50	4.8

**Table 3.** Calculated activation energy for charge-carrier hopping. [57] The value lies well below the thermal energy at room temperature (26 meV). Temperature-activated hopping alone, however, does not explain the transport behaviour at low temperatures

Thickness	σ_300K/_S cm^−1^	σ_0_/ S cm^−1^	E_a_(meV)
150 nm	1050	1800	10.88
400 nm	1050	1500	9.22

To obtain a proof-of-concept regarding our counter-ion hypothesis, drop-cast films (~6 µm thick) doped with gold(III)chloride and sulphuric acid were prepared and their conductivity was measured (Figure 4a, crimson and beige dots, respectively). The inset in Figure 3a focuses on their charge-transport in detail. These results cannot be directly compared to the results of PEDTT:sulf, however their internal comparison clearly suggests that sulphuric acid is the better choice of doping agent, especially in terms of conductivities at lower temperatures. Where the chloride-doped sample becomes insulating below 250 K, conductivities remained measurable in the acid-doped material down to 160 K. Further, the latter showed an initial increase of conductivity upon cooling. Since the chloride is the stronger oxidant in terms of the electrochemical series [48], the opposite would be expected, supporting our hypothesis of the effect of the counter-ion on disorder.

Since a flat contour of the conductivity plot suggests the presence of temperature-independent transport, it became of question in which phase of the metal–insulator transition (MIT) the material is found. The observation of metallic features and the presence of weak localization were of special interest to us. Therefore, we plotted our conductivity measurements at 0 and at 9 T in a so-called Zabrodskii- or W-plot [56] (Figure 4b). This kind of plot enhances even inconceivable changes in conductivity and consequently allows precise monitoring of the MIT. Crucial for this is the behaviour of the materials at low temperatures.

W was defined by Zabrodskii [58] amounting to following equation [57]:

$$W=\frac{\partial \ln\sigma }{\partial \ln T}$$

where *σ* is the conductivity and *T* the corresponding temperature.

Plotted against temperature (on logarithmic scales), three scenarios are possible:

A negative slope indicates thermally activated transport and is defined as an insulator; a positive one indicates a metallic material. The intermediate situation, *i.e*. where a flat contour is observed, is defined as the critical regime of the MIT.

In the absence of a magnetic field, the last situation was present (Figure 4d). Hence, PEDTT:sulf is intrinsically found in the critical state of the MIT. In this regime, computational fits for conductive behaviour were shown less reliable, and complex, as at low temperatures transport was dominated by electron–electron (e–e) interactions [58]. Under a strong magnetic field of 9 T, a phase transition to the insulating state was observed. Effects of weak localization were overruled as the magnetic field increased coherence among backscattered waves, indicating the prevalence of elastic scattering mechanisms [21,59–61].

This switching between conductive pathways was demonstrated in disordered metals, which exhibit similar transport behaviour also found in metallic polymers [32,58]. In the latter, an interplay of positive and negative effect of the magnetic field on conductivity would be expected. Our material was found in the critical regime of the MIT; therefore, it was of interest to conduct magnetoconductivity (MC) measurements at low temperatures to gain more insight in the transport mechanisms involved in the material. PEDTT:sulf sample thicknesses of 400 nm (Figure 4c,e) and 150 nm (Figure 4d,f) were prepared and measured for that purpose. Therein, fields of 0–9 T were applied at discrete temperatures below 10 K. As indicated by the conductive properties, positive MC was clearly observed in the thinner sample, only. In the thicker sample, the positive contribution, if present at all, was very weak and lost in the noise.

The positive MC in PEDTT:sulf was rather weak, with an increase of a mere 0.03%. Consequently, it could be argued that only a small fraction of the material actually underwent an increase in conductivity, indicating a portion of the material was in the metallic state, *i.e*. highly doped and devoid of disorder, embedded in non-metallic material [62]. This positive change was most pronounced for measurements at 1.9 K and 10 K. In the latter, two peaks were observed: 0.12 and 0.28 T. Whether this originated from a double resonance, *i.e*. two different conduction mechanisms being involved, spin-orbit effects [58,59], or from an overlap of two separate phases with different conduction mechanisms, remains subject to speculation. For temperatures between these, the positive MC was even less pronounced. The measurements at 3.9 K were especially puzzling, as no positive effect was observed. As the positive effect comes from weak localization, while the negative originates from electron–electron (e–e) interactions, the former must have been suppressed by either spin-orbit effects or by scattering on magnetic impurities [60,62]. Judging by the way the experiment was conducted; the latter seems like the more likely explanation.

The sample in question was first cooled to 1.9 K and subsequently exposed to a magnetic field ramped from 0 to 9 T. All further measurements were effectively subject to field cooling at 9 T, *i.e*. if the unpaired spins of the polymer would become collectively oriented in a permanent way by the magnetic field, they would remain so until a critical temperature is reached, where a phase-transition occurs and isotropic orientation is re-established [63,64]. Consequently, magnetic impurities would be generated that would result in the suppression of weak localization.

The above scenario is in agreement with the observed data, where at 3.9 K, only negative MC was observed in PEDTT:sulf, suggesting that weak localization is suppressed and e–e interactions dominate the charge-carrier transport. With rising temperature, the positive MC would become increasingly expressed as any residual magnetic features would break down above the critical transition temperature. This critical temperature was reached already at the next measurement step and a positive MC was observed. The phase transition is therefore expected to be found at a temperature between 3.9 and 6 K. Consequently, future studies must consider this type of polymer as a spin system switchable by magnetic fields rather than a plain conductive system. This would make PEDTT:sulf an interesting material to be investigated for its magnetic properties and in the long term could become of interest in the context of spintronics.

As we observed temperature independent transport, grain-boundaries do not limit charge-carrier transport and must be overcome, i.e. by polymers protruding from the crystals, with good π-orbital overlap to other polymer chains [9,65]. Hence, a 2D-type of transport and extensive presence of face-on oriented material is in the realm of possibility [9]. In PEDOT, the presence of thereof would be detectable in XRD as a peak between 25° and 28° 2θ and corresponds to a (0k0) orientation of the polymer [11,18,30].

**Figure 5.**
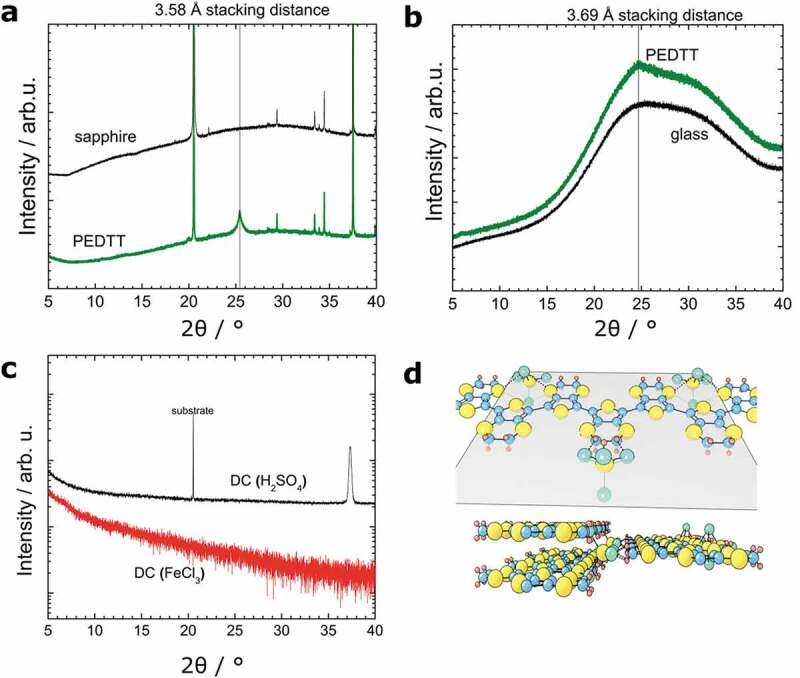
(a) XRD pattern of PEDTT sulphate on sapphire. 22–24 nm crystallites were found with a stacking of 3.58 Å. (150 nm sample thickness) (b) XRD pattern of PEDTT deposited on glass. A stacking distance of 3.69 Å was determined, indicating less disorder closer to the substrate. (150 nm sample thickness) (c) XRD pattern of drop-cast (DC) samples. The sulphate-containing sample shows a peak at 37.2°, while it was impossible to resolve any peaks on FeCl_3_ doped samples. (d) Based on the XRD-results and literature of PEDOT [28], we show a possible tertiary structure of PEDTT:sulf for illustration

Performing XRD experiments, indeed, a peak at 25.5° was found in measurements of PEDTT: sulphate deposited on top of sapphire (Figure 5a).

This peak was determined to correspond to a stacking distance of 3.58 Å, which is in good agreement with the value found for PEDOT:sulphate [32]. This value also coincides with the distance where the interaction between two sulphur atoms is the strongest, *i.e*. sulphurs van-der-Waals radius of 3.58 Å [38,66]. Hence, it is prudent to assume that this distance originates from PEDTT’s thioethers and represents the polymers stacking distance. This would mean that in our case, PEDTT-crystals are already as densely packed as physically possible and that this value would always be different from stacking distances found for PEDOT-species [12,17].

Further, we applied Scherrer’s formula assuming spherical particles [67]. The result indicated crystallites being 22–24 nm in diameter, which means that smaller crystals were formed as compared to PEDOT:sulphate generated by our group previously [21].

Additionally, PEDTT thin-films deposited on glass were measured by XRD (Figure 5b) and a stacking distance of 3.69 Å was determined. This distance is by 0.11 Å larger than for samples of sapphire. Because of the strong background signal of glass we refrain from drawing conclusions from this. However, it is well possible that we observe an effect of the substrate. This would suggest that the crystallites from which this signal originates are mainly situated in the vicinity of the substrate, which is highly interesting with recent developments in mind [9].

To support our hypothesis that the transition from amorphous to crystalline in PEDTT comes from the counter-ion and not the processing technique itself, drop-cast films doped with sulphuric acid and iron chloride were prepared (Figure 5c).

Despite a thorough scan, only the sulphuric acid doped material resulted in a peak at 37.2°. This corresponds to a crystallite size of 17–22 nm and a stacking distance of 2.4 Å. This is different from the peak found for PEDTT:sulf and is possibly linked to the difference between the thin-film and bulk film, such as that the (0k0) signal origins from a epitaxial effect of the sapphire substrate. Consequently, a different crystal form is induced, yet the result clearly shows the powerful influence of the counter-ion, even in drop-cast films.

For the above reason, we believe that a semi-crystalline material is achieved in PEDTT:sulf. This suggests that the structural effect of S–S repulsions in PEDTT were overcome by the introduction of a matching counter-ion and a situation unlike other PEDTT-species is observed [22,23,35]. In fact, an extended π -system is a necessity in this context, with well overlapping π-orbitals of the polymer, similar to PEDOT [23,28,32,52].

Consequently, we suggest a possible crystal structure, with a planar conformation of polymer chains with the hydrogen sulphate counter-ions intercalating in-between stacks. This is in good agreement with the model suggested by Massonnet et al. for PEDOT [52]. The geometries of the counter-ion would allow the small ion to be either in-plane with the polymer chain or to be found in-between two polymer plains, interacting with the ethylene via hydrogen bonds or with the sulphur found in the polymer [23] (Figure 5d).

## Conclusions

The combination of an optimized, reliable deposition technique (oCVD) with a careful choice of counter-ion delivered a PEDTT-based material with conductivities above 1000 S cm^−1^. This amounts of a drastic improvement in conductivity by >2600-fold. The high-performance with respect to charge-carrier transport is linked to quasi 2D transport related to polymer chains oriented face-on oriented with respect to the substrate.

A proof-of-concept using drop-cast films doped with sulphuric acid and strongly oxidative auric(III)chloride (or iron chloride for XRD measurements) supports the latter hypothesis as the chloride-doped samples resulted in lower conductivities and amorphous material.

PEDTT:sulf is found in the critical regime of the MIT yet exhibits positive magneto-conductivity effects and is possibly magnetisable. This was observed during MC-measurements where the measurement at 3.9 K did only exhibit negative MC, while the next measurement at 6 K showed a weak positive effect. This is most likely caused by scattering on magnetic impurities and asks for further investigation in terms of magnetic properties as it might lead to interesting applications in spintronics.

The material exhibits interesting optical properties in the visible region.

Interestingly, lower sample thicknesses resulted in higher conductivities, suggesting that face-on orientation of polymer chains and locally enhanced inter-chain transport similar to PEDOT is likely [9]. This opens up the question of how substrate-surface variation could be used to tune charge transport.

Most importantly, this paper raises the question, whether non-covalent interactions have received sufficient attention in conductive polymers. Theoretical investigations could lead to advancements in the field, which might innovate existing polymers, deliver completely new conductive systems, and develop strategies to optimize them for specific applications.
